# *In vitro* elimination of antimicrobials during ADVanced Organ Support hemodialysis

**DOI:** 10.3389/fphar.2024.1447511

**Published:** 2024-12-16

**Authors:** Christina König, Otto Frey, Susanne Himmelein, Lisa Mulack, Alexander Brinkmann, Aritz Perez Ruiz de Garibay, Tobias Bingold

**Affiliations:** ^1^ Department of Intensive Care Medicine, University Medical Center Hamburg-Eppendorf, Hamburg, Germany; ^2^ Center for Anti-Infective Research and Development, Hartford Hospital, Hartford, CT, United States; ^3^ Department of Pharmacy, General Hospital of Heidenheim, Heidenheim, Germany; ^4^ ADVITOS GmbH, Munich, Germany; ^5^ Department of Anesthesiology, Special Pain Management and Intensive Care Medicine, Heidenheim General Hospital, Heidenheim, Germany

**Keywords:** extracorporeal organ support, pharmacokinetics, antibiotic, renal replacement therapy, drug monitoring

## Abstract

**Background:**

Acute kidney injury (AKI) requiring continuous renal replacement therapy is common in critically ill patients. The ADVanced Organ Support (ADVOS) system is a novel hemodialysis machine that uses albumin enriched dialysate which allows the removal of protein-bound toxins and drugs. To date, data on antimicrobial removal under ADVOS has not yet been reported.

**Methods:**

An *in vitro* study was conducted using whole porcine blood and continuous infusions of different antimicrobial agents to investigate the effect of ADVOS on drug exposure. Drugs with varying protein binding, molecular weights and renal clearances, anidulafungin, cefotaxime, daptomycin, fluconazole, ganciclovir, linezolid, meropenem and piperacillin were studied.

**Results:**

All studied drugs were removed during the *in vitro* ADVOS experiment. Clearance under ADVOS (CL_ADVOS_) for low protein-bound drugs, such as cefotaxime, fluconazole, ganciclovir, linezolid, meropenem and piperacillin ranged from 2.74 to 3.4 L/h at a blood flow of 100 mL/min. With a doubling of flow rate CL for these drugs increased. Although efficiently removed, this effect was not seen for CL_ADVOS_ in high protein-bound substances such as daptomycin (1.36 L/h) and anidulafungin (0.84 L/h).

**Conclusion:**

The ADVOS system effectively removed protein-bound and unbound antimicrobials to a significant extent indicating that dose adjustments are required. Further, clinical studies are necessary to comprehensively assess the impact of ADVOS on antimicrobial drug removal. Until clinical data are available, therapeutic drug monitoring should guide antimicrobial dosing under ADVOS.

## 1 Introduction

Patients in the intensive care unit (ICU) who suffer from acute organ failure often require extracorporeal therapies to support failing organ systems (Huber and Ruiz de Garibay, 2020).

Moreover, infections are common in these patients and may trigger and/or worsen the progressive failure of one or more organ systems (Bajaj et al., 2014). Renal replacement therapy (RRT) for acute kidney injury (AKI), albumin dialysis, or plasma exchange for acute-on-chronic liver failure (ACLF) or acute liver failure (ALF) can be used to temporarily support failing organs. In RRT molecular size, protein binding, renal excreted fraction and volume of distribution are key determinants of drug removal, impacting under- or overdosing with risking adverse patient outcomes (Kollef et al., 2021). Additonally, studies have demonstrated that achieving appropriate antimicrobial exposure is crucial for reducing mortality rates in critically ill patients (Pai Mangalore et al., 2022; Roberts et al., 2014; Gatti et al., 2024). To guide antimicrobial dosing, *in vitro* studies (Roehr et al., 2015) as well as clinical trials investigating antimicrobial dosing under RRTs in critically ill patients (Roberts et al., 2020) have been published.

Among the profusion of extracorporeal organ replacement devices, the ADVanced Organ Support (ADVOS) albumin hemodialysis system (ADVITOS GmbH, Munich, Germany) has recently reported promising results in observational trials and is gaining attention for critically ill patients (Acharya et al., 2022; Sommerfeld et al., 2023). ADVOS is intended to remove water-soluble toxic substances, protein-bound toxic substances, to normalize or improve the composition of blood in case of e.g., electrolyte or acid-base disturbances (e.g., metabolic acidosis or respiratory acidosis). Additionally, it removes fluids in case of fluid overload in patients with acute, chronic and acute-on-chronic liver failure and/or renal failure and/or acidosis. However, studies investigating drug elimination under ADVOS have not been carried out systematically yet. Thus, understanding mechanistic and clinical factors that influence drug elimination is of major importance to attain appropriate drug exposure. Therefore, the present study employed a standardized *in vitro* blood model to assess the ability of ADVOS to remove a range of antimicrobial substances widely used in the ICU. Moreover, we aimed to quantify the amount of drug elimination to translate this information in clinical dosing regimens.

## 2 Materials and methods

### 2.1 *In vitro* model and blood preparation

Fresh porcine blood (Münchner Schlachthof Betriebs GmbH, Munich, Germany) was diluted with modified Ringer’s solution to achieve a hematocrit of 36%, standard electrolyte concentrations, and normal blood gas values. Porcine blood was mixed with human albumin (Albunorm 20%, Octapharma GmbH, Langenfeld, Germany) to obtain a human albumin level of 35 g/L. To prevent coagulation, 30,000 IU/L of heparin (Ratiopharm, Ulm, Germany) was added. The blood was maintained at a constant temperature of 37°C while continuously being stirred at 130 rpm. Drugs listed in Table 1 were individually added to attain initial concentrations as reported in Table 2.

**TABLE 1:** Study drugs including pharmacokinetic properties.

Drug	Manufacturer	Solvent	Protein binding [%]	Half-live [h]	V_d_ [L/kg BW]	CL_healthy_ [L/h]	Molecular size [Da]	Planed initial concentration [mg/L]
Anidulafungin	Pfizer, New York City, New York, US	R: SWFI D: 0.9% NaCl	99	40	0.5	1.08	1,140	8
Cefotaxime	Fresenius Kabi Deutschland GmbH, Bad Homburg, DE	R: SWFI D: 0.9% NaCl	33	1	0.3	16.54	455	100
Daptomycin	ratiopharm GmbH, Ulm, DE	R: 0.9% NaCl	90	8	0.1	0.61	1,620	140
Fluconazole	Fresenius Kabi Deutschland GmbH, Bad Homburg, DE	RTA	10	30	0.6	1.13	306	60
Ganciclovir	Hexal AG, Holzkirchen, DE	R: SWFI D: 0.9% NaCl	2	3	0.7	8.43	255	30
Linezolid	Fresenius Kabi Deutschland GmbH, Bad Homburg, DE	RTA	31	5	0.6	6.62	337	30
Meropenem	Fresenius Kabi Deutschland GmbH, Bad Homburg, DE	R: SWFI D: 0.9% NaCl	2	1	0.25	12.99	383	60
Piperacillin	Fresenius Kabi Deutschland GmbH, Bad Homburg, DE	R: SWFI D: 0.9% NaCl	16	1	0.25	12.99	518	164

SWFI: sterile water for infusion, NaCL: sodium chloride, RTA: ready to administer, R: reconstitution, D: dilution, Pharmacokinetic information is based on the prescribing information of each drug. Molecular sizes were taken from the PubChem Database.

**TABLE 2:** Drug concentrations and clearance results.

Drug	Infusion rate (mg/h)	Added drug (mg)	Eliminated drug (mg/9 h)	Drug removal (%)	CL_ADVOS_ [L/h]		CL_CRRT_ [L/h]
					BFR 100 mL/min CFR 160 mL/min DFR 800 mL/min	BFR 200 mL/min CFR 320 mL/min DFR 800 mL/min	BFR 200 mL/h DFR 2 L/h
Anidulafungin	5.1	63	38	61	0.84	0.84	0.02
Cefotaxime	74.9	849	787	93	2.74	4.77	1.34
Daptomycin	74	1,004	787	78	1.04	1.36	0.10
Fluconazole	56	576	540	94	3.39	6.20	1.80
Ganciclovir	23.7	262	246	94	2.91	5.88	1.98
Linezolid	26.8	281	258	92	2.79	4.65	1.38
Meropenem	59.2	595	556	93	3.40	6.08	1.96
Piperacillin	164	1784	1,667	93	3.18	5.61	1.56

Residual CL (renal + non-renal) is neglected for calculation purposes. CRRT, clearance was calculated assuming a dialysate flow rate of 2 L/h. BFR: blood flow rate; CFR: concentrate flow rate; CL: clearance; CRRT: continuous renal replacement therapy; DFR: dialysate flow rate; grey shaded rows indicate drug with protein binding >90%.

### 2.2 ADVOS hemodialysis system

**FIGURE 1:**
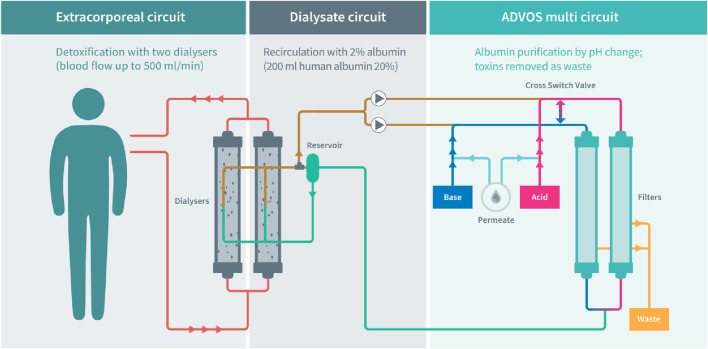
Schematic presentation of the ADVOS multi hemodialysis system. Adapted from https://advitos.com/wp-content/uploads/2023/07/20220630_ADVOS_kreislauf_poster_EN.pdf.

The ADVOS multi hemodialysis system (ADVITOS GmbH, Munich, Germany) utilizes three circuits (Figure 1) with two ELISIO 19H dialyzers (Nipro D. Med Germany GmbH, Hamburg, Germany) in a concurrent extracorporeal circuit. Blood flow rates (BFR) ranging from 100 to 500 mL/min are used with recirculating, albumin enriched (200 mL, 20% human albumin) dialysate at a flow rate of 800 mL/min (dialysate circuit) which employs acidic (Acid) and alkaline concentrates (Base) along with water for pH adjustments.

The dialysate is subsequently divided into an acidic and an alkaline path (ADVOS multi circuit). Herein, protein-bound and water-soluble toxins separate from the albumin dialysate through pH adjustment and convection. This arrangement effectively removes protein-bound molecules by inducing conformational changes in albumin, releasing bound molecules which are then removed through convection via two filters (ELISIO 11 H, Nipro D. Med Germany GmbH, Hamburg, Germany). Thereafter, the unloaded albumin dialysate is reintroduced into the dialysate circuit at a customized pH (Acharya et al., 2022). This conformation allows a continuous and efficient removal of protein-bound molecules as reported previously (Acharya et al., 2022; Sommerfeld et al., 2023; Huber et al., 2017; Falkensteiner et al., 2021). The concentrate flow rate (CFR) can range between 160 and 320 mL/min, determining both the quantity of dialysate cleaned via convective transport and the amount of fresh concentrate pumped into the system in the ADVOS multi circuit.

### 2.3 Experimental setup

**FIGURE 2:**
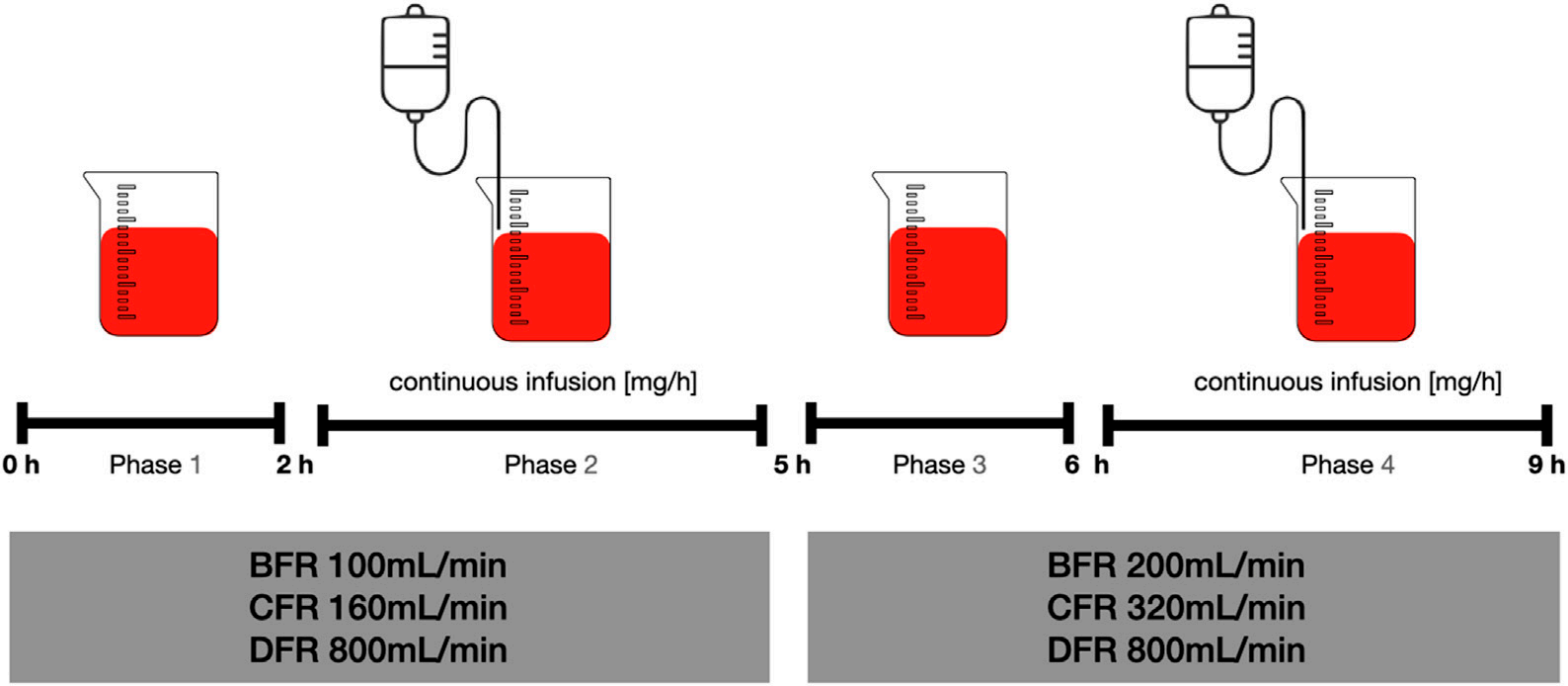
Consecutive phases of the experiment. BFR: Blood flow rate, CFR: Concentrate flow rate, DFR: Dialysate flow rate.

The experiment comprised various consecutive phases designed to facilitate pharmacokinetic profiling of each drug during ADVOS treatments (Figure 2). A 4-L blood beaker containing 35 g/L albumin (Albunorm 20%, Octapharma GmbH, Langenfeld, Germany) was spiked with each individual drug (Table 2). The initial drug concentrations were set to mimic physiological peak concentrations (Roehr et al., 2015).

In phase one, ADVOS multi was started with a BFR, CFR and dialysate flow rate (DFR) of 100, 160, and 800 mL/min, respectively. Sampling from the beaker stated at the beginning (T0) to assess starting concentrations with an additional sample at 2 h (T2).

Phase two was initiated by the start of continuous infusions for 3 h as outlined in Table 2 with subsequent sampling at 5 h (T5).

For phase three, BFR and CFR were doubled to 200 mL/min and 320 mL/min, respectively while DFR remained fixed (800 mL/min). Sampling was performed after 1 h (T6) followed by phase four, where continuous infusion was resumed and ceased with a final sample at T9.

Additional blood samples were collected from the tubing system of the machine (pre and post the two parallel dialyzers) at T0, T2, T5, T6 and T9 h.

#### 2.3.1 Drug quantification

The blood samples were centrifuged (4,000 rpm, 10 min), and the resulting serum supernatant separated into aliquots, which were stored at −80°C until further analysis. To obtain protein-free ultrafiltrate 800 µL serum was added to a Centrifree^®^ tube (Ultrafiltration device with Ultracel^®^ regenerated cellulose membrane, Merck KGaA, Germany) and spun at 1,000  ×  g, 30 min, 37°C. Subsequent quantification for both serum and ultrafiltrate was conducted using validated high performance liquid chromatography (HPLC) methods with UV detection. The methods already demonstrated linearity, with a relative standard deviation (SD) for intra- and inter-day precision and accuracy <10% at high, medium and low concentrations (Zimmer et al., 2021).

### 2.4 Pharmacokinetic calculations

Pharmacokinetic parameters of each drug were calculated through a non-compartmental analysis, assuming first-order kinetics and a volume of distribution of 4 L (beaker volume), utilizing Microsoft Excel 365 (Microsoft Corp., Redmond, WA, United States). A dialysate flow rate of 2 L/h was assumed for the calculation of CL_CRRT_ (Equation 2). This is based on a dialysis dose of 25 mL/kg/h for an 80 kg patient, as suggested by the KDIGO 2012 clinical practice guideline for patients with AKI requiring CRRT (Khwaja, 2012). ADVOS clearance (CL_ADVOS_) (Equation 1) and protein binding (PB) (Equation 3) were calculated as follows:

$${CL}_{ADVOS}\;\left[L/h\right]=\frac{\text{drug}\;\text{dose}\;\left[\frac{\text{mg}}{24\;h}\right]}{\text{concentration}\;\text{in}\;\text{serum}\;\left[\frac{\text{mg}}{L}\right]*24\;h\;}$$ (1)

$${CL}_{CRRT}\;\left[L/h\right]=\text{dialysate}\;\text{flow}\;\text{rate }\left[\frac{L}{h}\right]*\left(1-\text{protein}\;\text{binding}\right)$$ (2)

$$PB\;\left[\%\right]=100\;\%-\frac{\text{concentration}\;\text{in}\;\text{ultrafiltrate}\left[\frac{\text{mg}}{L}\right]}{\text{total}\;\text{concentration}\;\text{in}\;\text{serum}\;\left[\frac{\text{mg}}{L}\right]}\times 100$$ (3)

Dose (D) adjustments (Equation 5) were calculated using the Dettli equation (Equation 4) which determines the individual clearance (CL_individual_) based on the underlying renal function and the non-renal elimination fraction (Q_0_) (Dettli, 1974).

Remaining renal function was set to either a creatinine clearance (CrCL) of 25 mL/min or anuria (CrCL = 0 mL/min). CL_individual_ resembles the endogenous CL as it combines non-renal and residual renal elimination based on CrCL.

$${CL}_{individual}\left[L/h\right]=Q_{0}+\frac{\text{creatinine}\;\text{clearance}\;\text{of}\;\text{the}\;\text{patient}\;\left[\frac{\text{ml}}{\min}\right]}{100\;\frac{\text{ml}}{\min}}*\left(1-Q_{0}\right)*{\text{CL}}_{\text{healthy}}\;\left[\frac{L}{h}\right]$$ (4)

The individually required dose (D) without ADVOS was calculated by proportionally adjusting CL_individual_ to CL_healthy_, as reported in the literature (see Table 1), and multiplying it by the standard licensed dose.

$$D\;\left[mg/24h\right]=\frac{{\text{CL}}_{\text{individual}}\left[\frac{L}{h}\right]}{{\text{CL}}_{\text{healthy}}\;\left[\frac{L}{h}\right]}*\text{standard}\;\text{dose}\;\left[\frac{\text{mg}}{24h}\right]$$ (5)

The required dose using ADVOS (D_ADVOS_) was calculated by adding the CL_ADVOS_ from our experiments to CL_individual_ (Equation 6).

$$D_{ADVOS}\left[mg/24h\right]=\frac{{\text{CL}}_{\text{individual}}\left[\frac{L}{h}\right]+{\text{CL}}_{\text{ADVOS}}\;\left[\frac{L}{h}\right]}{{\text{CL}}_{\text{drug}}\;\left[\frac{L}{h}\right]}*\text{standard}\;\text{dose }\left[\frac{\text{mg}}{24h}\right]$$ (6)

Visualization and statistical evaluation were performed using linear or non-linear regression methods included in Prism 9 (GraphPad Software, San Diego, CA, United States).

## 3 Results

### 3.1 Protein binding *in vitro*

**FIGURE 3:**
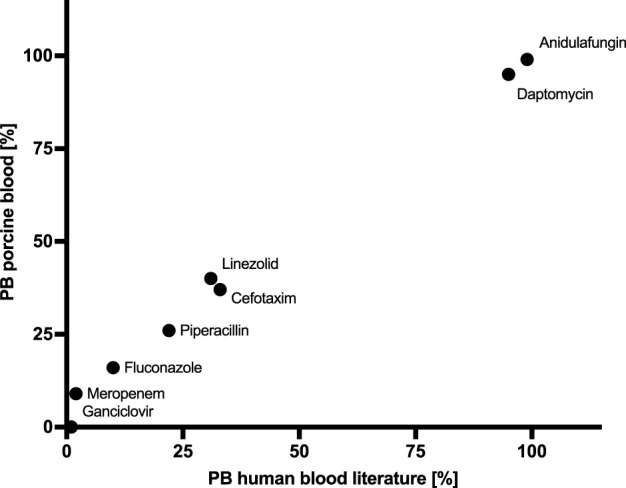
Protein binding (PB) *in vitro* vs. literature.

Protein binding were comparable to those found in the literature (Figure 3). As the free fraction of ganciclovir was below the limit of quantification (LLOQ) protein binding was calculated using ½ of the LLOQ.

### 3.2 Observed drug concentrations and clearance during ADVOS treatments

**FIGURE 4:**
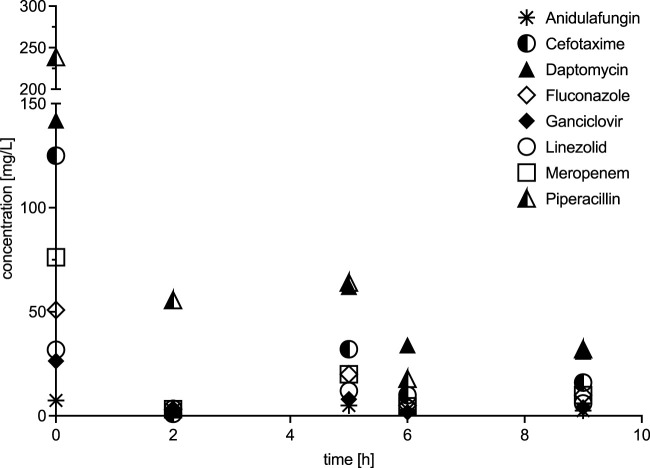
Concentration profiles over time.

All drugs showed a decline in concentrations (Figure 4). At a BFR of 100 mL/min, low protein-bound drugs with a low molecular weight, such as cefotaxime, fluconazole, ganciclovir, linezolid, meropenem and piperacillin showed a CL_ADVOS_ ranging from 2.74 to 3.4 L/h. An increase in the BFR to 200 mL/min resulted in a doubling of CL_ADVOS_ for all the aforementioned drugs.

For the two drugs with the largest molecular weight and highest protein binding daptomycin and anidulafungin CL_ADVOS_ at a BFR of 100 mL/min were 0.84 and 1.06 L/h, respectively. Doubling the BFR resulted in a moderate increase in CL_ADVOS_ only for daptomycin (1.36 L/h).

### 3.3 Calculated dose adjustment for ADVOS treatments in patients with impaired renal function

Dose calculations based on the CL_CRRT_ and on the CL_ADVOS_ under the two different settings of ADVOS therapy are shown in Table 3. Drugs with a high protein binding ≥90% such as anidulafungin and daptomycin as well as linezolid with a moderate protein binding revealed an extended CL_ADVOS_ in the *in vitro* experiment (Table 2) resulting in increased daily dosages when compared to the licensed maintenance dosages for patients with CrCL >60 mL/min. In contrast, the low protein-bound (<35%) beta-lactam antibiotics such as cefotaxime, meropenem and piperacillin as well as the antiviral agent ganciclovir showed a significantly lower CL_ADVOS_ (Table 2), compared to patients with normal renal function (CL_healthy_, Table 1). Thus, lower recommended doses were calculated since the cumulated CL_individual_ at CrCL ≤25 mL/min and CL_ADVOS_ was not as high as CL_healthy_ for those drugs. In the case of fluconazole, at a blood flow of 100 mL/min CL_ADVOS_ triplicates (Table 2) the CL_healthy_ (Table 1), which results in higher required doses during ADVOS treatments (Table 3). Finally, the calculated dosages under CRRT were lower than under ADVOS for each of the drugs (Table 3).

**TABLE 3:** Dose calculations based on *in vitro* CL_ADVOS_ and calculated CL_CRRT_.

Treatment	No extracorporeal support			ADVOS				CRRT	
Settings				BFR 100 mL/min CFR 160 mL/min DFR 800 mL/min		BFR 200 mL/min CFR 320 mL/min DFR 800 mL/min		BFR 200 mL/h DFR 2 L/h	
CrCL (mL/min)	>60	25	0	25	0	25	0	25	0
Dose calculations (mg/24 h)	Standard licensed dose								
Anidulafungin	100	100	100	180	180	180	180	100	100
Cefotaxime	6,000	3,080	2,100	4,070	3,090	4,810	3,830	3,560	2,590
Daptomycin	700	440	360	1,630	1,550	2000	1920	550	470
Fluconazole	800	380	240	2,780	2,640	4,770	4,630	1,660	1,520
Ganciclovir	800	260	80	540	360	820	640	450	270
Linezolid	1,200	930	840	1,440	1,350	1770	1,680	1,180	1,090
Meropenem	3,000	1,310	750	2,100	1,540	2,720	2,150	1770	1,200
Piperacillin	12,000	6,420	4,050	8,640	6,540	10,880	8,780	7,150	5,040

CrCL: creatinine clearance; BFR: blood flow rate; CFR: concentrate flow rate; DFR: dialysate flow rate; grey shaded rows depict drugs requiring higher dosages under ADVOS, compared to standard licensed drug regimens.

## 4 Discussion

To date, several cohort studies as well as data from a patient registry (Fuhrmann et al., 2021; Fuhrmann et al., 2020) demonstrate effective removal of water-soluble and protein-bound substances with ADVOS (Huber et al., 2017; Falkensteiner et al., 2021; Kaps et al., 2021). Although each of these reports describes the therapy as feasible and safe, data regarding pharmacokinetics of anti-infective agents were not presented. Our study is the first to systematically investigate the effect of ADVOS on the elimination of antimicrobials. The results highlight that all tested drugs were efficiently removed by the ADVOS system. Moreover, blood and concentrate flows played a role in the removal of small water-soluble drugs as CL_ADVOS_ for these drugs doubled with doubling of flow rates. Small molecules with low protein binding (<35%) showed pronounced removal, with CL_ADVOS_ ranging from 2.74 L/h (cefotaxime) up to 3.4 L/h (meropenem) at a BFR of 100 mL/min, CFR of 160 mL/min and a fixed DFR of 800 mL/min. The doubling of the CL_ADVOS_ for low protein-bound drugs with a twofold increase in the BFR (200 mL/min) and CFR (320 mL/min) during the experiment strengthens this assumption. These data correlate well with CL_ADVOS_ (6.3 L/h at BFR 250 mL/min) published in a clinical case report (König et al., 2021).

Our data showed that, assuming a standard dialysate flow of 2 L/h for CRRT, CL_ADVOS_ surpassed calculated CL_CRRT_ for all tested anti-infective agents (Table 2). Moreover, CL_ADVOS_ was higher than previously reported clinical total body clearances for patients receiving different types of CRRT. For instance, meropenem CL_ADVOS_ was up to 6.08 L/h, whereas studies report lower total body clearances under CRRT (4.1 L/h (Varghese et al., 2015); 3.5 L/h (Bilgrami et al., 2010); 1.9 L/h (Jamal et al., 2015)). The same trend was observed for linezolid (CL_ADVOS_ 4.65 vs. 2.3 L/h (Meyer et al., 2005)) and piperacillin (CL_ADVOS_ 5.61 vs. 2.1 L/h (Sharrock et al., 2023) or 3.9 L/h (Awissi et al., 2015)). Moreover, Muhl et al. reported total fluconazole clearance under CRRT of 2.57 L/h (Muhl, 2005), which was well exceeded when using ADVOS (CL_ADVOS_ up to 6.2 L/h). For ganciclovir, a similar pattern was seen with a reported total body clearance under CRRT of 2.71 L/h (Horvatits et al., 2014), in comparison to a CL_ADVOS_ of 5.88 L/h. Interestingly, this effect was not as pronounced for cefotaxime, which showed CRRT clearance of 5.8 L/h (Koedijk et al., 2016), whereas CL_ADVOS_ was up to 4.77 L/h. Overall, the high removal rates are in line with using two parallel dialyzers (total surface area 3.8 m^2^) in combination with high CFR (160–320 mL/min) which can explain the high CL_ADVOS_ observed for antimicrobials with low protein binding.

For larger molecules with protein binding >90% such as daptomycin or anidulafungin, minor or no changes in CL_ADVOS_ were observed with increased flows. Daptomycin showed low clearance (0.56 L/h (Xu et al., 2017)) with negligible changes in pharmacokinetics and clearances in patients undergoing CRRT (CL 1.1 L/h) (Bellmann and Smuszkiewicz, 2017) whereas CL_ADVOS_ is more than twice as high (1.36 L/h). Anidulafungin presented with an *in vitro* CL_ADVOS_ of 0.84 L/h (Table 2), which is almost the non-renal excretion capacity of healthy volunteers (Wasmann et al., 2018). In this scenario, the total clearance under ADVOS is twice as high as in healthy volunteers (CL_healthy_, Table 1). Therefore, highly protein-bound drugs would require higher maintenance dosages under ADVOS to achieve optimal targets (Table 3). In this experiment, the removal of high protein-bound antimicrobials is likely due to the utilization of an albumin primed dialysate, which is continuously regenerated (ADVOS multi circuit). However, the recirculation of the dialysate might hinder a complete removal of circulating substances.

There are certain limitations that need to be discussed. We report an *in vitro* experiment designed to investigate the elimination capacity of the ADVOS hemodialysis system, as reported previously (Roehr et al., 2015; Konig et al., 2019; Wenzler et al., 2022). Herein, similar to other studies characterizing drug removal by extracorporeal support we substituted human with porcine blood (Schneider et al., 2021). Yet, its results cannot be translated into direct clinical practice as pathophysiological changes affecting antimicrobial exposure in critical illness were absent in this approach. In critically ill patients, metabolic and biochemical changes, such as hypoalbuminemia, can alter drug ionization and protein binding (Wong et al., 2018). Furthermore, resuscitation strategies and vasoactive medications can induce a hyperdynamic state, increasing blood flow to major organs and affecting hepatic metabolism and renal excretion (Roberts et al., 2011). Due to the frequent use of multiple medications in critically ill patients, drug-drug interactions further influence metabolism and pharmacokinetic exposure [ (Koeck et al., 2021). These factors contribute to both inter- and intra-patient variability in drug pharmacokinetics, making drug response difficult to predict in this population (Roberts and Lipman, 2009). Moreover, our experiment did only simulate for a single dosing interval and conducted over a limited time course (9h). Therefore, reduced ADVOS effectiveness over the course of therapy (24h) cannot be ruled out. Thus, these data can be used to guide future clinical studies to identify appropriate dosing regimens whilst using ADVOS. Extrapolation to other substance classes often used in critical illness settings should be used cautiously but might help to inform clinicians to closely monitor therapies in terms of efficacy. Further studies are warranted to investigate and validate the findings of this *in vitro* experiment. In the meantime, therapeutic drug monitoring (TDM) of antimicrobials is highly recommended to guide and optimize treatment in critically ill patients undergoing ADVOS treatment.

## 5 Conclusion

Our study shows for the first time that the ADVOS hemodialysis system can efficiently remove antimicrobials. In comparison to CRRT, low protein-bound drugs follow a similar removal mechanism under ADVOS, which is dependent on BFR and dialysis dose (i.e., concentrate flow). However, the CL_ADVOS_ appeared higher than CL calculated and/or reported under CRRT. Moreover, in contrast to CRRT, ADVOS removed protein-bound antimicrobials (e.g. anidulafungin, daptomycin) with clearances comparable to those in healthy volunteers. Until clinical data are available, individualized dosing and TDM are recommended to guide effective and safe concentrations under ADVOS treatments.

## Data Availability

The raw data supporting the conclusions of this article will be made available by the authors, without undue reservation.
